# Review of the genus *Urgleptes* Dillon (1956) of Hispaniola (Coleoptera, Cerambycidae, Acanthocinini): descriptions of five new species and one new synonymy

**DOI:** 10.3897/zookeys.532.6587

**Published:** 2015-11-05

**Authors:** Ian S. Ravin, Steven W. Lingafelter

**Affiliations:** 1 Natural History Research Experience Program, Department of Entomology, National Museum of Natural History, Washington D.C. 20013–7012, U.S.A; 2 Systematic Entomology Laboratory, Agriculture Research Service, U.S. Department of Agriculture, National Museum of Natural History, Washington D.C. 20013–7012, U.S.A

**Keywords:** Longhorned beetle, endemism, intraspecific variation, taxonomy, systematics, synonymy, new species

## Abstract

The genus *Urgleptes* Dillon (1956) is reviewed for Hispaniola. Five new species of *Urgleptes* from the Dominican Republic are described herein: *Urgleptes charynae* Ravin & Lingafelter, **sp. n.** (La Vega province), *Urgleptes conjunctus* Ravin & Lingafelter, **sp. n.** (Peravia Prov.), *Urgleptes curtipennis* Ravin & Lingafelter, **sp. n.** (Independencia Prov.), *Urgleptes marionae* Ravin & Lingafelter, **sp. n.** (Monseñor Nouel Prov.), and *Urgleptes obliteratus* Ravin & Lingafelter, **sp. n.** (Pedernales Prov.). Two additional, previously described species are newly recorded for Hispaniola: *Urgleptes puertoricensis* Gilmour and *Urgleptes sandersoni* Gilmour. It is established that *Urgleptes haitiensis* Gilmour is a new synonym of *Urgleptes sandersoni*. Thus there are seven species of *Urgleptes* recorded for Hispaniola. For all species photographs, illustrations, full descriptions, distribution maps, and a dichotomous key are included for their identification.

## Introduction

The genus *Urgleptes* is among the largest in the Acanthocinini (Lamiini) with 79 described species (Monné 2015). Of these, 11 species were described from the Caribbean region. These include: *Urgleptes borikensis* Micheli and Micheli (2004) from Puerto Rico, *Urgleptes chamaeropsis* (Fisher, 1926) from Cuba and the Bahamas, *Urgleptes clarkei* Chemsak (1966) from Peter Island and Antigua, *Urgleptes cobbeni* Gilmour (1963) from the Lesser Antilles, Curaçao, Bonaire, and Barbados, *Urgleptes gahani* Chalumeau (1983) from St. Vincent, *Urgleptes guadeloupensis* (Fleutiaux & Sallé, 1889) originally described from Guadeloupe, and reported from throughout the Lesser Antilles and Greater Antilles, *Urgleptes haitiensis* Gilmour (1963) from Haiti, *Urgleptes hummelincki* Gilmour (1968) from Aruba, *Urgleptes leopaulini* Touroult (2004) from Guadeloupe, Martinique, and Saint Lucia, *Urgleptes puertoricensis* Gilmour (1963) from Puerto Rico, and *Urgleptes sandersoni* Gilmour (1963) from Puerto Rico (Monné 2015; Tavakilian and Chevillotte 2015).

*Urgleptes* Dillon (1956) is very similar to the even larger genus, *Lepturges* Bates (1863). Species of *Urgleptes* have distinct punctures limited to a row along the basal transverse sulcus extending behind the lateral pronotal spines (Fig. 8e). In *Lepturges*, punctures along the basal transverse sulcus do not extend behind the lateral pronotal spines and there are other scattered, large punctures on the pronotal disk (Dillon 1956; Lingafelter 2007).

Results of these studies demonstrate that seven species of *Urgleptes* occur in Hispaniola. These include five endemic species described as new herein: *Urgleptes charynae*, *Urgleptes conjunctus*, *Urgleptes curtipennis*, *Urgleptes marionae*, *Urgleptes obliteratus*, and two formerly described species that represent new country records for the Dominican Republic: *Urgleptes puertoricensis* and *Urgleptes sandersoni*. We provide justification for the new synonymy of *Urgleptes haitiensis* with *Urgleptes sandersoni*.

## Materials and methods

The material consulted in this study is primarily the result of expeditions from the Smithsonian Institution (second author), Carnegie Museum (John Rawlins, Robert Androw, and Robert Davidson), Florida State Collection of Arthropods (Paul Skelley and Michael Thomas), and The West Indian Beetle Fauna Project (Michael Ivie), along with the individual collections of Edmund Giesbert, Kelvin Guerrero, Charyn Micheli, Julien Touroult, Robert Turnbow, James Wappes, Norman Woodley, and many others. Acronyms for collections consulted herein are listed below:

ACMT American Coleoptera Museum San Antonio, TX, USA (J. Wappes)

CMNH Carnegie Museum of Natural History, Pittsburgh, PA, USA (J. Rawlins, R. Davidson, R. Androw)

FSCA Florida State Collection of Arthropods, Gainesville, FL, USA (E. Giesbert, P. Skelley, M. Thomas)

RHTC Robert H. Turnbow, Jr. Private Collection, Ft. Rucker, AL, USA

SWLC Steven W. Lingafelter Private Collection, North Potomac, MD, USA

USNM Smithsonian Institution, Washington, DC, USA (S. Lingafelter)

WIBF West Indian Beetle Fauna Project, Bozeman, MT, USA (M. Ivie)

Holotypes of new species described herein are deposited in the USNM and maintained on the online image database of Lingafelter, et al. (2015) following the standards and methods of Lingafelter, et al. (2014). Label data are mostly verbatim, but redundancy among paratypes is minimized by not repeating identical localities from specimens from the same institution. Specimen data are grouped in alphabetical order by provinces (which are not repeated for brevity). Haiti records are listed after Dominican Republic Records. Species description format follows Lingafelter and Micheli (2009).

Specimens were photographed with a Zeiss AxioCam HRc camera attached to a Zeiss Discovery.V20 stereomicroscope™ with a PlanApo™ 0.63X objective and Dolan-Jenner MI-150 Fiber Optic Illuminators™ with gooseneck fiber optic and ring light attachments. Image stacking was achieved by a combination of Axiovision™ and Adobe Photoshop CS6™ software.

## Results and discussion

### Urgleptes puertoricensis

Taxon classificationAnimaliaColeopteraCerambycidae

Gilmour, 1963

Figs 1 , 8b , 15

#### Diagnosis.

This species can be distinguished easily by the mostly dark brown scape, slightly flavous at the base (in *Urgleptes sandersoni* the scape is darkened apically with a subapical pale ring). This species is most similar to the highly variable *Urgleptes sandersoni* by the elytral maculae (most specimens have a darkened periscutellar region and a zigzag pubescent fascia extending from the lateral margin obliquely toward the middle combined with variable subapical maculae). The posthumeral macula never attains the base in *Urgleptes puertoricensis*, whereas in *Urgleptes sandersoni* this macula originates basally and extends along the epipleuron.

**Figures 1–2.**
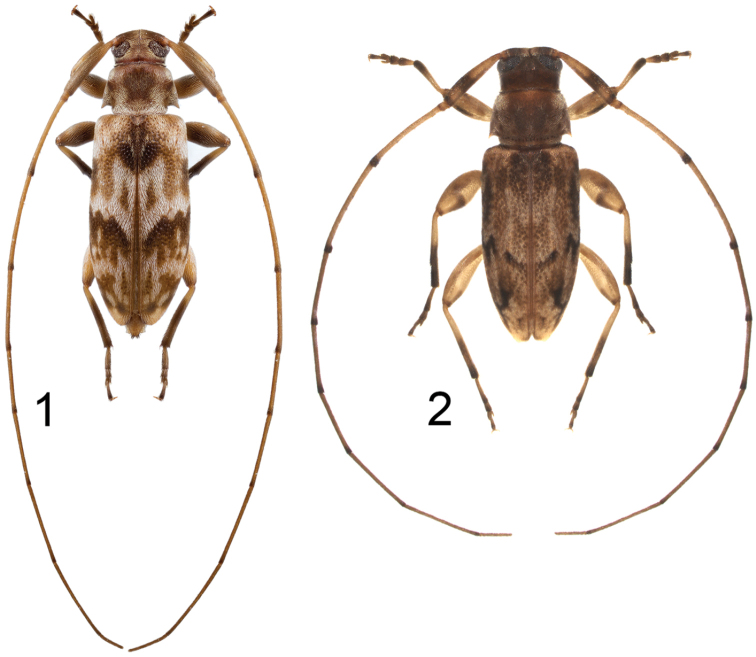
Dorsal habitus photographs of described species of *Urgleptes*. **1** *Urgleptes puertoricensis* Gilmour **2** *Urgleptes sandersoni* Gilmour.

#### Redescription.

*Measurements*: body length: 3.1–4.7 mm; body width: 1.1–1.5 mm; elytral length: 2.1–3.2 mm; elytral width: 0.6–0.8 mm; pronotal length: 0.6–0.9 mm; pronotal width: 1.0–1.4 mm; body length/pronotal length: 4.9–5.1; elytral length/elytral width: 3.7–4.1; pronotal length/pronotal width: 0.6.

*Head*: covered in moderately dense, ashy, appressed setae, becoming denser on genae and eye margins; setae light brown at vertex suture. *Antenna*: with exception of apical antennomeres being mostly dark, antennomeres 3–7 flavous, covered with moderately dense, appressed, dark pubescence; dark annulate at apices. Antennal apices with thickened bristle-like setae along mesal surface of third and fourth segments. Antennae extending beyond elytral apices by 4–6 segments. Antennal scape basally flavous, transitioning to brown; covered in fine gold pubescence apically. Scape extending to posterior one-fourth of pronotum. *Eye*: lower eye lobe about twice the height of upper eye lobe; lobes connected by 5–7 rows of ommatidia in most specimens. Upper eye lobes separated by little more than greatest width of scape. *Mouthparts*: frontoclypeal margin with two long, black setae aligned with edge of upper eye lobe. Clypeus without pubescence; labrum pallid with sparse, translucent setae; and 4–6 long, suberect, black setae two-thirds length of those found at frontoclypeal margin.

*Thorax*: pronotum broadly rounded at sides to posteriorly directed, short, acute tubercle on posterior fourth of each side; constricted along posterior fifth behind lateral tubercles; constriction demarcated with row of large, separate punctures across disc, continuing behind base of tubercles and down sides (partially obscured by dense white pubescence); no other distinct punctures visible. Dense, white anterolateral pubescence converging posteromedially around brown pubescent fascia of variable width. Lateral tubercles covered in dense, white pubescence extending ventrally to proepimeron. No distinct calli present on pronotal disc. Pronotal integument mostly brown but darker beneath brown pubescent fascia along posterior constriction and base of lateral tubercles; anterior pronotal collar distinctly dark brown. Prosternum smooth, impunctate, covered with uniform, translucent pubescence. Prosternal process narrow between procoxae (less than one-fifth width); greatly expanded behind procoxae. Mesosternum smooth, impunctate, covered with uniform, translucent pubescence. Mesosternal intercoxal process slightly broader than prosternal process, just less than one-fourth width of mesocoxa. Metasternum smooth, impunctate, covered with uniform, appressed, ashy-white to translucent pubescence. Integument of ventral sclerites mostly light brown, becoming dark brown at sides. Scutellum brown with nearly black posterior edge (some specimens posteriorly fringed with white pubescence); broadly rounded posteriorly. *Elytra*: moderately densely, coarsely punctate (some specimens with punctures more pronounced than others); punctures partially obscured by dense, appressed, mottled pubescence; elytra lacking tubercles and erect setae. Integument mostly light brown, with exception of darker brown maculae and corresponding dark brown pubescent fasciae. Periscutellar region dark brown to black (sometimes with medial, white pubescence parallel to suture). This region often surrounded by light brown integument covered in dense, white pubescence reaching slightly variable dark brown submedial epipleural macula that is mostly covered in white and brown pubescence. Elytron with dark macula as follows: pubescent zigzag fascia extending from lateral margin obliquely towards middle, not quite attaining suture; with two posteriorly directed parallel fasciae (occasionally weakly connected), ending postmedially along elytral disc. Subapical brown macula extending anterolaterally, narrowly branching toward suture (some specimens with branched maculae merged, almost connecting with medial maculation), surrounded by appressed, dense, white pubescence. Elytral apex subtruncate, with outer apical angle more produced posteriorly than sutural angle. *Legs*: femora mostly dark brown with exception of metafemora; slightly flavous basally and subapically, most apparent on metafemora; uniformly covered with pale white to gold pubescence. Tibiae basally flavous, becoming dark brown apically; with thickened postmedial bristle-like setae. Mesotibiae with dorsal concavity apically also lined with bristle-like setae. Tibiae approximately equal in length to femora. Tarsomeres basally flavous, becoming dark brown apically; coated with short, suberect, dark setae and off-white pubescence ventrally on second through fifth segments.

*Abdomen*: ventrites covered with appressed, pale to translucent pubescence. Integument light brown with posterior third of each ventrite noticeably darkened. Fifth ventrite one and one-half times longer than fourth ventrite and fringed with slightly denser off white pubescence.

#### Distribution.

This species was originally described from Puerto Rico and has also been recorded from the Virgin Islands (Monné 2015). Hispaniola and the Dominican Republic represent new island and country records, respectively, for this species. This species is uncommonly collected but wide ranging (Fig. 15) throughout the Dominican Republic. Most specimens have been collected by beating vegetation from May through July.

#### Material examined.

**DOMINICAN REPUBLIC: Barahona Prov.**, South slope of Sierra Martin Garcia, 530 m, 18°21.012'N, 71°01.765'W, 9 December 2014, S. W. Lingafelter (USNM, 2); **La Altagracia Prov.**, Parque Nacional del Este, Boca de Yuma entrance, 18°21.904'N, 68°37.087'W, beating vegetation at night, 5 August 1999, M. A. Ivie (WIBF, 2); **La Vega Prov.**, 1 km NW Manabao, 5 June 1994, M. C. Thomas (FSCA); **Monte Cristi Prov.**, 8 km N Villa Elisa, 31 May 1994, M. C. Thomas (FSCA); 13.2 km N Villa Elisa, 2 June 1994, M. C. Thomas (FSCA); **Pedernales Prov.**, 25 km N of Cabo Rojo, 679 m, 18°06.769'N, 71°37.245'W, day collecting, 10 July 2004, Charyn J. Micheli (USNM); Parque Nacional Jaragua, trail to Carlitos, 6 km S of hwy 44, 106 m, 17°48.93'N, 71°28.27'W, beating vegetation, 8 July 2004, Charyn J. Micheli (USNM, 2); Los Tres Charcos, 13 July 1996, M. C. Thomas (FSCA); **PUERTO RICO.**, Guánica Forest, 25–26 July 1969, H. & A. Howden (WIBF, 3).

### Urgleptes sandersoni

Taxon classificationAnimaliaColeopteraCerambycidae

Gilmour, 1963

Figs 2 , 8a, h , 9 , 16

Urgleptes haitiensis Gilmour, 1963: 82, syn. n.

#### Diagnosis.

The elytral maculae of this species are more variable than any other species in the genus. As discussed above, *Urgleptes sandersoni* is most likely to be confused with *Urgleptes puertoricensis*, but they can be distinguished most easily by the scape that is apically darkened with a subapical, flavous ring in *Urgleptes sandersoni* (mostly dark brown, without a subapical, pale ring in *Urgleptes puertoricensis*.) The presence of dark brown to black maculae at the base of the humeri, often extending through the epipleuron (only occurring posthumerally in *Urgleptes puertoricensis*) may also be used to differentiate between the two. The primarily Lesser Antillean *Urgleptes guadeloupensis* (Fleutiaux & Sallé) is much paler and lacks a marmorated pubescent pattern on the elytra (see Remarks for further discussion of this species).

#### Redescription.

*Measurements*: body length: 3.3–6.3 mm; body width: 1.2–2.3 mm; elytral length: 2.3–4.6 mm; elytral width: 0.6–1.2 mm; pronotal length: 0.6–1.0 mm; pronotal width: 1.0–1.7 mm; body length/pronotal length: 5.3–6.1; elytral length/elytral width: 4.0; pronotal length/pronotal width: 0.6–0.7.

*Head*: covered in fine vestiture of golden or pale brown appressed hairs; denser along eye margins and antennal tubercles; relatively sparse on occiput and anterior margin of frons. Narrow median-frontal line mostly visible, extending from frons through vertex, and posteriorly to occiput. Frontal-genal line extending from anterior tentorial pits along anterior margin of genae to base of mandible; mostly obscured by dense, golden, appressed pubescence. *Antenna*: mostly pale brown; covered with short, appressed, fine pubescence, not obscuring surface. Antennomeres 1–8 with distinct, narrowly dark brown apices; scape also with second, diffusely dark annula at apical two-thirds; antennomeres 9–11 vaguely darkened apically. Antennae considerably longer than body; extending beyond elytral apices by 4–5 antennomeres; scape extending to posterior margin of pronotum. *Eye*: Lower eye lobe over twice height of upper eye lobe; extending over half distance between antennal tubercle and frontal margin; lobes connected by 3–4 rows of ommatidia at narrowest point, in most specimens. Upper eye lobes separated by 1.5 times greatest width of scape. *Mouthparts*: frontoclypeal margin sparsely pubescent; lacking pubescent fringe; clypeus without pubescence except for few long setae originating at base. Labrum sparsely pubescent, with 8–10 long, suberect, translucent setae.

*Thorax*: pronotum broadly rounded at sides to posteriorly directed, short, acute tubercles on posterior fourth; constricted along posterior fifth behind lateral tubercles; constriction demarcated with row of large, separate punctures across disc, continuing behind base of tubercles, down sides; pronotum otherwise impunctate except for uniform micropunctures visible only under high magnification. No distinct calli present on disc. Pronotum with uneven vestiture of golden pubescence concentrated at sides of disc, around lateral tubercles, and, often, in two anteromedial spots on disk; elsewhere, pubescence is sparse. Integument mostly light brown, becoming darker at middle of disc, often extending to anteromedial and posteromedial margins. Prosternum smooth, impunctate, covered with uniform, appressed, golden or translucent pubescence. Prosternal process narrow between procoxae (less than one-sixth width) and greatly expanded behind procoxae. Mesosternum smooth, impunctate, covered with uniform, appressed, golden or translucent pubescence. Mesosternal intercoxal process broader than prosternal process, separating mesocoxae by about one-fourth width of mesocoxa. Metasternum smooth, impunctate, covered with uniform, appressed, golden or translucent pubescence. Integument of ventral sclerites mostly light brown, becoming dark brown at sides. Scutellum dark brown with mixture of dark, translucent, and golden pubescence; broadly rounded posteriorly. *Elytra*: densely, coarsely punctate; coated with moderately dense, appressed pubescence; with mottled light and dark appearance; lacking tubercles and erect setae. Highly variable in distribution of maculae and pubescence, often with a correspondence between integumental color and setal color. Nearly all specimens with very dark brown to black periscutellar region (occasionally extending posteriorly along most of suture). This region often surrounded by lighter brown integument covered in gray or translucent pubescence. Nearly all specimens with an oblique narrow post-medial macula extending from near the sutural margin anterolaterally toward lateral margin (sometimes complete, sometimes extending only one-third to one-half across elytral disk). This black macula bordered by white or gray setae. Humeri slightly projecting anterolaterally, usually with black or very dark macula at base and often poorly-demarcated epipleural region. Elytral apex obliquely truncate; lacking spines. *Legs*: femora mostly light yellowish-brown with variably sized dark maculae on mesal and lateral face (most pronounced on mesofemora; least pronounced on metafemora); femora mostly uniformly pubescent with combination of fine, appressed, white, translucent, and dark brown setae. Tibiae light yellowish-brown at base, dark brown to piceous at apical half to two-thirds. Tibiae with setae suberect and more stout, particularly. Mesotibiae with dorsal concavity lined with stout, dark, bristle-like setae. Tarsi, with exception of base of first segment, mostly dark brown to piceous on most specimens.

*Abdomen*: ventrites covered with fine, appressed, golden and translucent pubescence; light brown, becoming darker on basal segments and sides. Fifth ventrite distinctly longer than fourth in both sexes; with weak middle notch fringed by longer setae.

#### Distribution.

This species was originally described from Puerto Rico. We record it from the Dominican Republic (new country record). This is the most widespread and common species on the island of Hispaniola, having been collected at nearly every sampled locality (Fig. 16). We also record this species for Jamaica (new country record) and Navassa Island which lies between Jamaica and Hispaniola. Specific locality data are included for those islands below.

#### Remarks.

*Urgleptes sandersoni* and *Urgleptes haitiensis* were each described in Gilmour (1963) on the basis of one specimen; therefore, he was unable to assess intraspecific variation when he defined both species. He stated for *Urgleptes haitiensis* that it differs from *Urgleptes sandersoni* “in being of a general much darker colour and its marmorated appearance”, but shares the pale annulate antennal scape. We have studied hundreds of specimens from Hispaniola and Puerto Rico and found that this is a single, variable species. We demonstrate that the elytral markings vary tremendously (Fig. 9) and without a geographical basis. Figure 9a most closely matches the holotype of *Urgleptes sandersoni* (described from Puerto Rico). Figure 9f most closely matches the holotype of *Urgleptes haitiensis* (described from Haiti). The scape coloration, however, with the presence of the pale apical annulation in an overall darker apical half, is constant for the species as is the elytral punctation and general coloration of the femora, tibiae, and tarsi, described above. We therefore consider *Urgleptes haitiensis* Gilmour (1963: 82) **a new synonym** of *Urgleptes sandersoni* Gilmour (1963: 79). This species is most similar to *Urgleptes guadeloupensis* (Fleutiaux & Sallé), another highly variable species described from Guadeloupe. According to Gilmour (1963), that species is much paler without a marmorated appearance. Some authors (e.g., Peck 2005) have recorded *Urgleptes guadeloupensis* from the Greater Antilles (Cuba) but other authors (e.g, Micheli 2010) concluded it was not present in Puerto Rico. We agree with Micheli’s concept of *Urgleptes sandersoni*; however, further study and discovery of additional characters may support synonymy of *Urgleptes sandersoni* under *Urgleptes guadeloupensis*.

#### Material examined.

**DOMINICAN REPUBLIC: Barahona Prov.**, 11 km S Barahona, 6–17 May 1985, E. Giesbert (FSCA, 7); Filipinas, 1700 ft, 3–6 May 1985, E. Giesbert (FSCA); same data but 5–6 May 1985 (FSCA); Rd to Polo, S slope, 860 m, 14 July 1996, M. C. Thomas (FSCA, 34); 4.5 km S Barahona, 13 July 1996, M. C. Thomas (FSCA); Paraiso Río Nazaito, 18°00'N, 71°06'W, blacklighting/log picking, 7 July 2004, S. W. Lingafelter (USNM, 2); same data but Charyn J. Micheli (USNM, 3); Filipinas, 625 m, 18°07.339'N, 71°07.152'W, blacklighting/night beating, 7 July 2004, S. W. Lingafelter (USNM, 5); same data but 6 July 2004, C. J. Micheli (USNM); same data but 7 July 2004 (USNM); El Cachote, 970 m, 18°03.295'N, 71°09.778'W, beating, 14 July 2006, S. W. Lingafelter (USNM, 6); Rd to Filipinas, 1700 ft, V–15–1985, J. E. Wappes (WIBF); 11 km S Barahona, V–6–1985, J. E. Wappes (WIBF, 4); same data but V–15, 17–1985 (WIBF); **Dajabón Prov.**, El Pomo Loma de Cabrera, 366 m, 19°26'09.3"N, 71°34'45.3"W, beating/dead wood, 28 June 2010, S. W. Lingafelter (USNM); Los Cerezos, 14 km NW of Río Limpio, 608 m, 19°18'42.9"N, 71°36'36.6"W, fresh cut wood, 29 June 2010, S. W. Lingafelter (USNM, 10); **Elías Piña Prov.**, Sierra de Neiba, 1.5 km E of Military Post 204, SW of Aniceto Martinez, 1597 m, 18°41.644'N, 71°46.457'W, 12 July 2006, S. W. Lingafelter (USNM); Río Limpio, 867 m, 19°14'03.9"N, 71°31'00.9"W, MV lights, 30 June 2010, S. W. Lingafelter (USNM); **Hato Prov.**, Parque Nacional Los Haїtises, 6 km SW Sabana de la Mar, 10–20 m, 19°03.494'N, 69°27.302'W, beating, 6 July 2005, C. J. Micheli (USNM, 2); same data but S. W. Lingafelter (USNM, 2); **Independencia Prov.**, Rd 47 between Los Pinos and Ángel Félix, 760 m, 18°36.986'N, 71°46.556'W, 20 June 2005, N. E. Woodley (USNM, 2); **La Altagracia Prov.**, Parque Nacional del Este, Guaraguao, 0–5 m, 18°19.568'N, 68°48.500'W, day collecting, 21 July 2004, C. J. Micheli (USNM); same data but beating, S. W. Lingafelter (USNM); same data but 3 July 2006 (USNM); same data but 8 July 2006 (USNM); same data but 28 June 2005, on *Conocarpus erectus*, N. E. Woodley (USNM); same data but day collecting (USNM); Parque Nacional del Este, Boca de Yuma, 20 m, 18°21.508'N, 68°36.956'W, day collecting, 19 July 2004, C. J. Micheli (USNM, 2); same data but 20 July 2004 (USNM); El Verón, Hoyo Azul, 25–40 m, 18°33.610'N, 68°26.881'W, day collecting, 22 July 2004, C. J. Micheli (USNM, 3); same data but S. W. Lingafelter (USNM, 5); El Verón, Hoyo Azul, 25–40 m, 18°33.805'N, 68°26.543'W, 4 July 2005, N. E. Woodley (USNM); same data but day collecting, 1 July 2005, C. J. Micheli (USNM, 8); same data but 4 July 2005 (USNM, 2); same data but 1 July 2005, S. W. Lingafelter (USNM, 7); same data but UV light and log picking, 7 July 2006 (USNM, 3); Parque Nacional del Este, Boca de Yuma, 20 m, 18°21.508'N, 68°36.956'W, blacklight, 18 July 2004, S. W. Lingafelter (USNM); same data but day collecting, 19 July 2004 (USNM); Punta Cana near Ecological Reserve, 0–5 m, 18°30.477'N, 68°22.499'W, day collecting, 3 July 2005, S. W. Lingafelter (USNM, 5); same data but blacklight (USNM); same data but 29 June 2005 (USNM); same data but beating, 14 June 2005 (USNM, 8); same data but 12–14 June 2005 (USNM); same data but lights, N. E. Woodley (USNM, 4); same data but 2–7 July 2005 (USNM, 2); same data but 12–13 June 2005 (USNM, 5); same data but day collecting, 3 July 2005, C. J. Micheli (USNM, 9); same data but at lights, 26–27 July 2005 (USNM); Punta Cana near Ecological Reserve, 0–5 m, 18°30.477'N, 68°22.499'W, beating, 2 July 2006, S. W. Lingafelter (USNM, 5); same data but 21 July 2006 (USNM, 5); same data but cut wood at night, 5 July 2006 (USNM, 11); same data but at light, 1 July 2006 (USNM); Parque Nacional del Este, valle de la Orqueta, 25 m, 18°22.945'N, 68°46.631'W, beating, 29 June 2005, C. J. Micheli (USNM); same data but N. E. Woodley (USNM); **La Vega Prov.**, vicinity of Manabao, 15 July 1996, M. C. Thomas (FSCA, 6); 10 km E Constanza, 1295 m, beating in pine guava forest, 31 August 1988, M. A. Ivie, T. K. Philips, & K. A. Johnson (FSCA); 1 km NW Manabao, 6 July 1994, M. C. Thomas (FSCA, 4); same data but 4 July 1994 (FSCA, 2); same data but 5 July 1994 (FSCA); Pico Duarte Trail, Ciénaga to Los Tablones, 19°08.222'N, 70°27.736'W, beating, 29 June 2004, C. J. Micheli (USNM, 2); same data but 3000 ft, S. W. Lingafelter (USNM); same data but 3300 ft, 17 July 2004 (USNM); same data but recently fallen tree, 2 July 2010, S. W. Lingafelter (USNM, 2); Parque Nacional José Armando Bermúdez, 10 km along trail W of La Ciénaga near Los Tablones, 990–1100 m, 19°01.753'N, 70°54.654'W, day collecting, 22 June 2005, S. W. Lingafelter (USNM); Rancho Baiguate Hotel, 527 m, 19°07'36"N, 70°38'39"W, dead branches, 18 June 2010, S. W. Lingafelter (USNM, 5); 9 km NE Jarabacoa, 2000 ft, 8–12 May 1985, E. Giesbert (FSCA, 8); Boca de Yuma entrance, Parque Nacional del Este, 12 m, 18°201.904'N, 68°37.094'W, beating vegetation, 6 August 1999, M. A. Ivie (FSCA); same data but 2 m, at light, 5 August 1999, M. A. Ivie & K. A. Guerrero (FSCA); 6 km E Jima, 4100 ft, 18 May 1985, E. Giesbert (FSCA); 10 km NE Jarabacoa, V–8, 12–1985, J. E. Wappes (WIBF, 5); 2 km E Tireo, 4100 ft, V–8–1985, J. E. Wappes (WIBF); **Monseñor Nouel Prov.**, 19°01'N, 70°31'W, beating, 4 July 2004, S. W. Lingafelter (USNM); **Monte Cristi Prov.**, Reserva Cientifica Villa Elisa, 183 m, 19°44'46.1"N, 71°15'27.3"W, dead wood, 24 June 2010, S. W. Lingafelter (USNM, 4); **Pedernales Prov.**, 19–20 km N Cabo Rojo, 375 m, 10 July 1996, M. C. Thomas (FSCA, 4); same data but 24 km N, 535 m, 11 July 1996 (FSCA); N of Pedernales border Rd, Río Banano, S of Arroyos, 18°09.291'N, 71°45.540'W, 21 July 1999, Ivie & Guerrero (FSCA); Parque Nacional Sierra de Baoruco, Las Abejas, 1240 m, 18°09.023'N, 71°37.387'W, 09 August 1999, M. A. Ivie (FSCA); Parque Nacional Jaragua, trail to Carlitos ca. 6 km S of hwy 44, 106 m, 17°48.93'N, 71°28.27'W, beating, 8 July 2004, C. J. Micheli (USNM, 7); same data but S. W. Lingafelter (USNM, 4); same data but 16 June 2006 (USNM, 4); Parque Nacional Sierra de Baoruco, Las Abejas, 1150 m, 18°09.011'N, 71°37.342'W, blacklight, night beating, 11 July 2004, S. W. Lingafelter (USNM, 14); same data but 18 June 2005 (USNM, 2); same data but 17 July 2006 (USNM, 10); same data but beating, 11 July 2004, Charyn J. Micheli (USNM, 9); 25 km N of Cabo Rojo, 679 m, 18°06.769'N, 71°37.245'W, day collecting, 10 July 2004, S. W. Lingafelter (USNM, 2); 1 km N of Banano, 290 m, 18°09.258'N, 71°45.384'W, day beating, 12 July 2004, S. W. Lingafelter (USNM); 25 km N of Cabo Rojo, 679 m, 18°06.769'N, 71°37.245'W, beating, 15 July 2006, S. W. Lingafelter (USNM); Parque Nacional Jaragua 3 km S of Los Tres Charcos, 100 m, 17°47.51'N, 71°27.78'W, beating, 16 July 2006, S. W. Lingafelter (USNM, 6); **Peravia Prov.**, 5 km W of Rd to El Río, S of Pedregal, 52 m, 19°05.092'N, 70°35.864W’, 23 June 2005, S. W. Lingafelter (USNM, 3); **Puerto Plata.**, Reserva Cientifica Isabel de Torres, 704 m, 19°45'55.6"N, 70°42'42.8"W, beating, 23 June 2010, S. W. Lingafelter (USNM, 9); S of Punta Rusia, 39 m, 19°49.32.5"N, 71°13'11.1"W, dead wood, S. W. Lingafelter (USNM, 2); S Pico Isabel de Torres, El Cupey Rd, 258 m, 19°45.214'N, 70°43.6464'W, 30 July 1999, Ivie & Guerrero (FSCA); 14 km W Puerto Plata, 10–11 May 1985, E. Giesbert (FSCA, 2); 14 km W of Puerto Plata, V–11–1985, J. E. Wappes (WIBF); Le Cumbre Rsh. Sta., V–8, 9–1985, J. E. Wappes (WIBF); Imbert, at night, 29 July 1983, R. E. Woodruff (FSCA); **San Pedro Prov.**, Near Juan Dolio, V–13, 18–1985, J. E. Wappes (WIBF, 17); **San Pedro de Macorís Prov.**, 12 km W San Pedro de Macorís, 5–19 May 1985, E. Giesbert (FSCA, 10). **HAITI: Dept. Ouest.**, Morne Guimby, 22 km SE Fonds-Verrettes, 6500 ft, 19 July 1956, B. & B. Valentine (WIBF, 8). **Dept. Sud- Est.**, Massif de la Selle, Morne d’Enfer, 1850 m, 15 May 1984, M. C. Thomas (FSCA, 4); Parc National La Viste, 1 km S Roche Plat, 22 May 1984, M. C. Thomas (FSCA, 4): Furcy (Holotype of *haitiensis*).

### Urgleptes charynae

Taxon classificationAnimaliaColeopteraCerambycidae

Ravin & Lingafelter sp. n.

http://zoobank.org/C6C1795B-4F4F-4C5A-BC97-2D6C64D3F497

**Figures 3–7.**
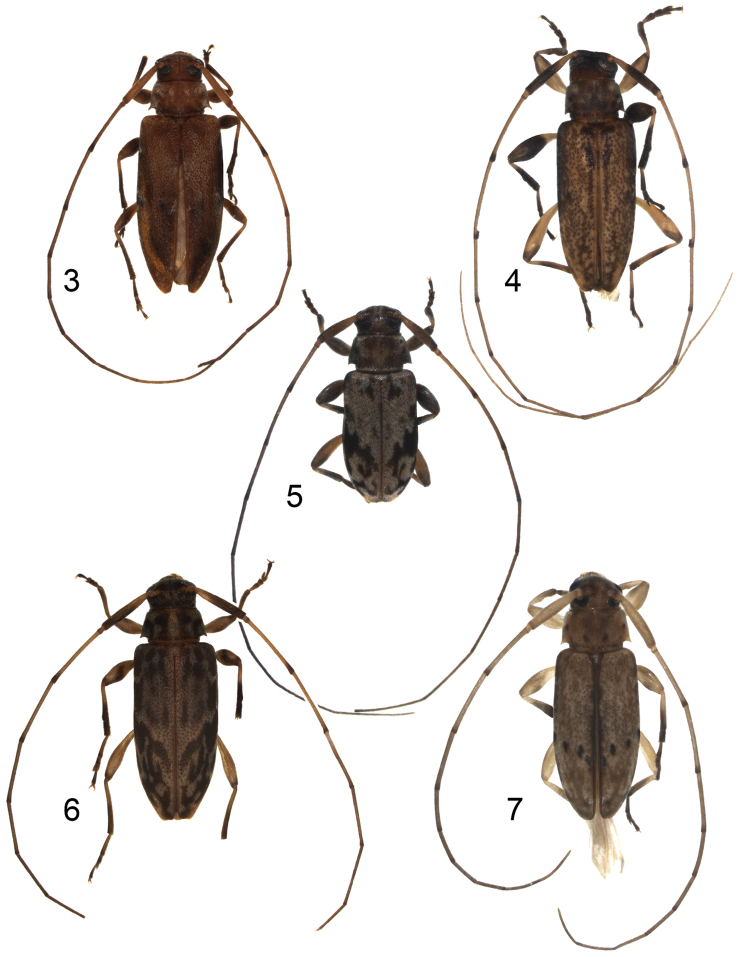
Dorsal habitus photographs of new species of *Urgleptes*. **3** *Urgleptes charynae* Ravin and Lingafelter **4** *Urgleptes conjunctus* Ravin & Lingafelter **5** *Urgleptes curtipennis* Ravin and Lingafelter **6** *Urgleptes marionae* Ravin & Lingafelter **7** *Urgleptes obliteratus* Ravin & Lingafelter.

**Figure 8.**
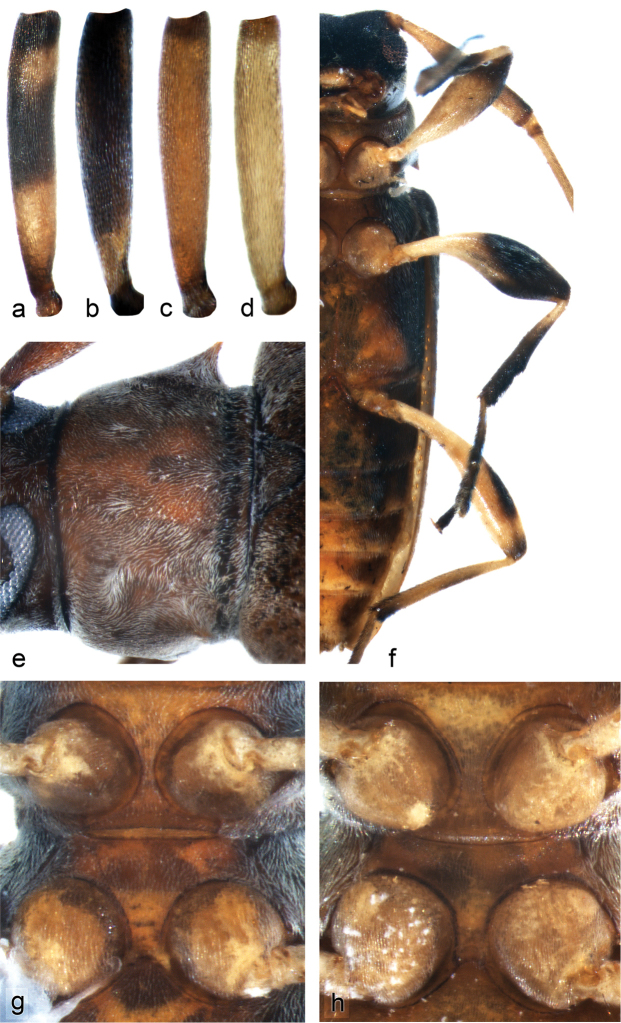
Morphological feature of *Urgleptes* species. **a–d** Scapes of *Urgleptes*: **a** *Urgleptes sandersoni* **b** *Urgleptes puertoricensis* **c** *Urgleptes charynae* **d** *Urgleptes obliteratus* **e** Detail of posterior row of pronotal punctures characteristic of the genus and dense pronotal pubescence concentrated around lateral tubercles in *Urgleptes charynae* **f** Ventral view of *Urgleptes conjunctus* emphasizing distinctly dark mesofemora and mesotibiae and very narrow procoxal and mesocoxal processes **g** Narrow procoxal process and broad mesocoxal process for *Urgleptes marionae* **h** Broad procoxal and mesocoxal process for *Urgleptes sandersoni*.

Figs 3 , 8c , 10 , 17

#### Diagnosis.

This species is recognized by the mostly uniform, light ferruginous integument covered by mostly uniform pubescence. The elytra have slightly denser patches of white pubescence postmedially extending across the disk (sometimes forming circular patches). Underneath these pubescent patches, the integument is slightly darkened. The pronotum has dense, white pubescence anterolaterally, extending to the lateral tubercles. The only other species with mostly uniform integument color and mostly lacking dense pubescent patches is *Urgleptes obliteratus*. That species is easily distinguished by its much paler integument and typically bold, black, postmedial elytral and pronotal spots. Additionally, *Urgleptes charynae* has the scape uniformly brown, slightly darker apically, unlike other species which usually contain a subapical spot or flavous base.

**Figure 9.**
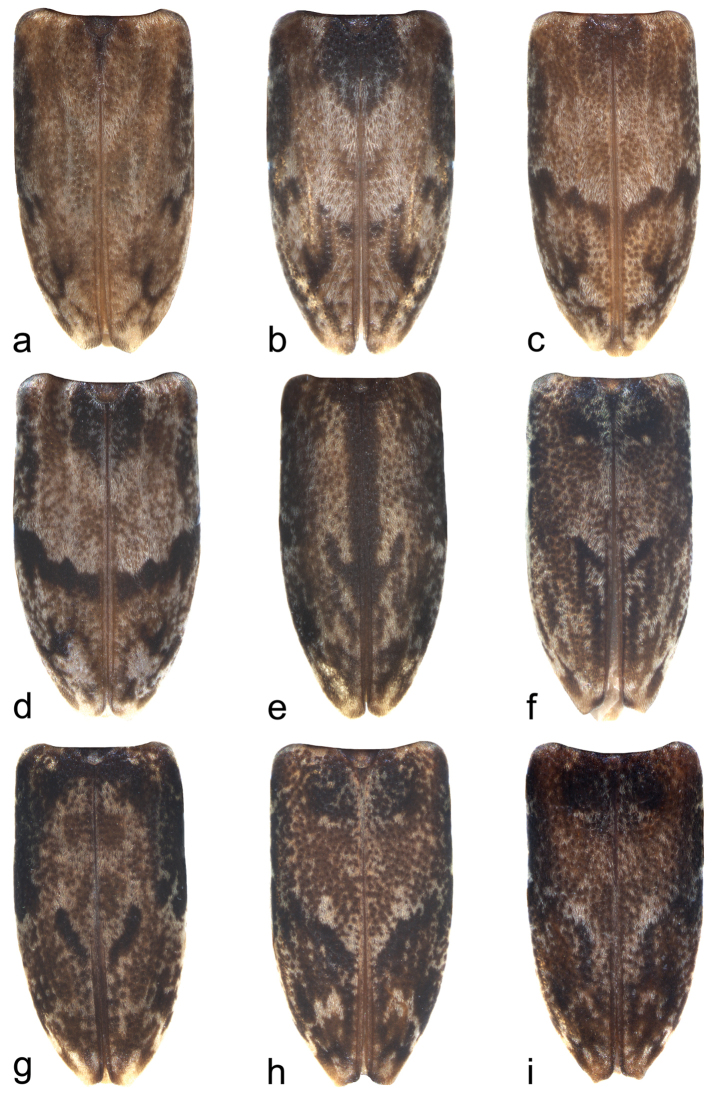
Morphological series showing major elytral maculae for *Urgleptes sandersoni* and its new synonym, *Urgleptes haitiensis*. **a** Pattern most similar to *Urgleptes sandersoni* holotype **f** Pattern most similar to *Urgleptes haitiensis* Gilmour holotype.

**Figure 10.**
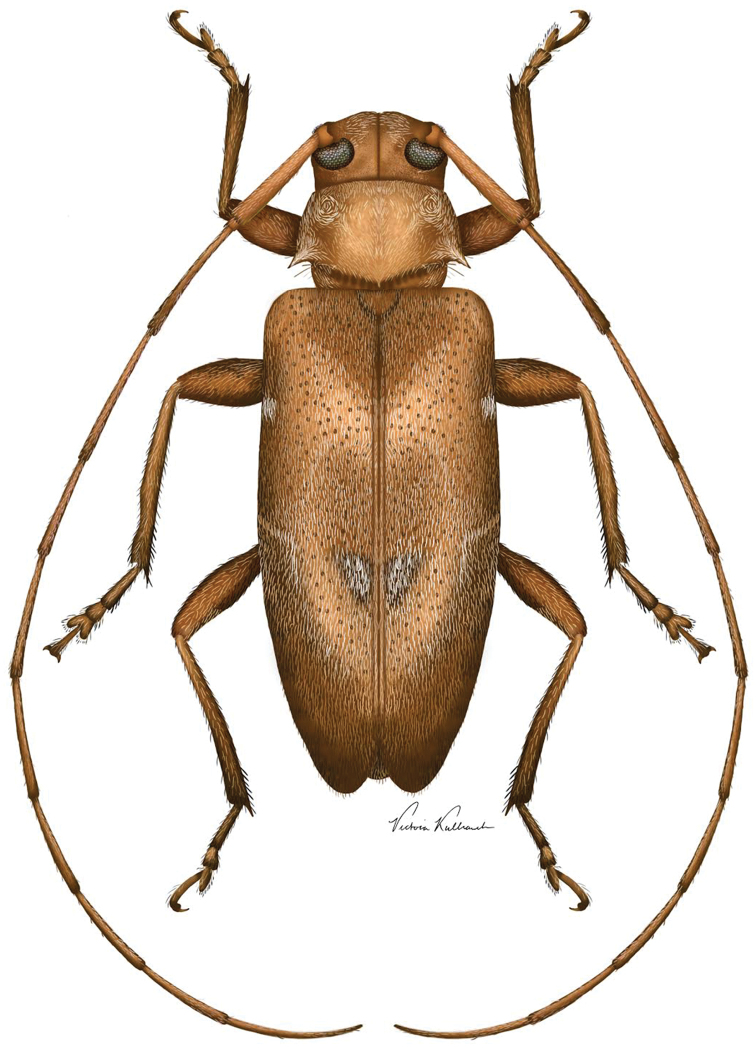
Digital painting of *Urgleptes charynae* sp. n. (illustrated by Victoria Kulhanek, St. Paul, Minnesota).

#### Description.

*Measurements*: body length: 3.6–5.3 mm; body width: 1.3–1.9 mm; elytral length: 2.5–3.8 mm; elytral width: 0.7–1.0 mm; pronotal length: 0.7–1.0 mm; pronotal width: 1.1–1.6 mm; body length/pronotal length: 5.3–5.4; elytral length/elytral width: 3.9–4.0; pronotal length/pronotal width: 0.6.

*Head*: integument brown, becoming darker at frontoclypeal margin, and apices of antennal tubercles. Covered in vestiture of appressed white pubescence on genae, occiput, and posterior margin of lower eye lobe. *Antenna*: with exception of apical antennomeres being uniform throughout, antennomeres mostly flavous with darkened apices; covered in dense appressed, gold to brown pubescence. Antennal apices with thickened bristle-like setae along mesal surface of third and fourth segments of most specimens. Sixth antennomere shortest, being just under three-fourths length of third antennomere. Antennae extending beyond elytral apices by approximately 5 segments. Antennal scape extending to posterior fourth of pronotum; uniformly light brown, weakly darker at apex; covered in uniform, appressed, gold pubescence. *Eye*: lower eye lobe slightly more than 2× height of upper eye lobe; lobes narrowly connected by about 5 rows of ommatidia in most specimens. Upper eye lobes separated by slightly more than greatest width of lower eye lobe. *Mouthparts*: clypeus pallid; lacking distinct pubescence; and finely punctate. Labrum slightly darker than clypeus; with 7 suberect, dark brown setae; anteriorly fringed with translucent setae. Mandibles light brown, distinctly darkened on apical fourth; with two dark brown setae along lateral margin.

*Thorax*: pronotum broadly rounded at sides to posteriorly directed, short, acute tubercles on posterior fourth; constricted along posterior fifth behind lateral tubercles; constriction demarcated with row of large, separate punctures across disc, continuing behind base of tubercles and down sides; no other distinct punctures visible. Integument mostly brown, slightly darker medially and along posterior constriction; anterior pronotal collar distinctly dark brown. Pronotal disc finely coated in gold pubescence; with anterolateral patches of white pubescent fasciae; surrounding pubescence dense, ashy-white, covering tubercles; sometimes concentrating on posterior constriction behind lateral spines. No distinct calli present on pronotal disc. Prosternum impunctate; covered in dense vestiture of white pubescence. Prosternal process extremely narrow between procoxae, about half width of anterior pronotal collar, and greatly expanded posteriorly. Mesepimeron toward metepisternal apex coated with dense, appressed, white pubescence becoming thinner ventrally. Mesosternal intercoxal process about twice as broad as prosternal process. Integument of ventral sclerites mostly light brown, with margin of coxal cavity darkened. Scutellum brown with darker posterior edge; broadly rounded posteriorly. *Elytra*: moderately dense, coarsely punctate; elytral disc mostly light-ferruginous; dark brown at epipleuron; dark brown postmedially to apex; covered in mottled white-grey to brown pubescence. Periscutellar region slightly raised; coated in ferruginous pubescence; surrounding region weakly concave; with moderately dense, appressed, white-grey pubescence extending from humerus to submedial suture. Epipleural integument mostly dark brown; medially giving rise to discontinuous, oblique, dark brown maculae, each surrounded by distinctly dense, white, appressed, pubescent fasciae. Postmedial pubescence along disc white-grey; integument becoming dark brown to apical third. Elytral apex subtruncate, with outer apical angle slightly more produced posteriorly than sutural angle. *Legs*: femora mostly light-ferruginous; slightly darker laterally, most visibly on mesofemora; uniformly covered with pale-golden pubescence. Tibiae basally flavous-ferruginous; submedially dark brown towards apices with dark brown, postmedial, thickened, bristle-like setae. Mesotibiae with dorsal concavity apically, also lined with bristle-like setae. Tibiae slightly longer than femora; hind legs slightly longer than forelegs. Tarsomeres basally flavous-ferruginous, becoming brown apically; generally coated with short, suberect, dark setae; with off-white ventral pubescence on lobed fourth tarsomere.

*Abdomen*: ventrites covered with moderately dense, appressed, pale pubescence; integument mottled brown, posteriorly lighter, apical margin pallid. Fifth ventrite one and a half times longer than fourth ventrite and fringed with slightly denser golden pubescence.

#### Distribution.

Endemic to Hispaniola, this species is known from both the Cordillera Central and southern Sierra de Neiba mountain ranges (Fig. 17) where it has been collected from May through November, at high elevations between 600–2450 m, often in or near pine forests.

#### Etymology.

We name this species for our friend and colleague, Charyn Micheli, who collected the holotype and many other specimens in the genus. The epithet is a noun in apposition.

#### Type material.

**Holotype** (male): **DOMINICAN REPUBLIC: La Vega Prov.**, Pico Duarte trail below La Compartición, 2450 m, 19°02.254'N, 70°58.155'W, day collecting, 1 July 2004, Charyn J. Micheli (USNM); **Paratypes: DOMINICAN REPUBLIC: La Vega Prov.**, 10 km NE of Jarabacoa, 8–12 May 1985, J. E. Wappes (ACMT); Sierra de Neiba, 9.4 km SSW El Cercado, 1973 m, 18°39'18"N, 71°32'51"W, hand collected in meadow near mature pine forest, 18–19 November 2004, C. W. Young, J. E. Rawlins, C. Nunez, V. Verdecia, and W. Zanol (CMNH, 2); 9 km NE of Jarabacoa, 2000 ft, 8–12 May 1985, E. Giesbert (FSCA); 9 km NE of Jarabacoa 8–12 May 1985, E. Giesbert (FSCA, 2); Constanza, 17 July 1996, R. Turnbow (RHTC, 2); 13 km S of Constanza, El Convento Rd, 1450 m, 18°50.915'N, 70°41.059'W, 28 July 1999, M. A. Ivie & K. A. Guerrero (WIBF); 3 km N of Jarabacoa, 25 May 1992, R. Turnbow (RHTC, 2); 1 km N of Tireo Arriba, 24 May 1992, R. Turnbow (RHTC); 1 km NW Manabao, 6 June 1994, M. C. Thomas (FSCA); 1.4–2.6 km E of Manabao, 5 June 1994, M. C. Thomas (FSCA, 5); 2.6–6.4 km E of Manabao, 4 June 1994, M. C. Thomas (FSCA); Vicinity of La Cienaga, mercury vapor/UV light, 18 July 1996, M. C. Thomas (FSCA, 3); 1 km NW Manabao 6 June 1994, M. C. Thomas (FSCA); 1.4–2.6 km E of Manabao, 6 June 1994, M. C. Thomas (FSCA); **San Juan Prov.**, San Juan, 7 km N of Arroyo Cano, 1 km S of Los Fríos, 1120 m, 18°52'N, 71°01'W, second growth pine forest, 1 September 1995, J. Rawlins, G. Onore, R. Davidson (CMNH); **Santiago Prov.**, Parque Nacional José Armando Bermúdez, El Rodeo, 1456 m, 19°08'N, 71°02'W, 10 July 1992, M. A. & R. O. Ivie (WIBF).

### Urgleptes conjunctus

Taxon classificationAnimaliaColeopteraCerambycidae

Ravin & Lingafelter sp. n.

http://zoobank.org/3334A9B6-0BAB-49A0-95FA-018F86497253

**Figure 11.**
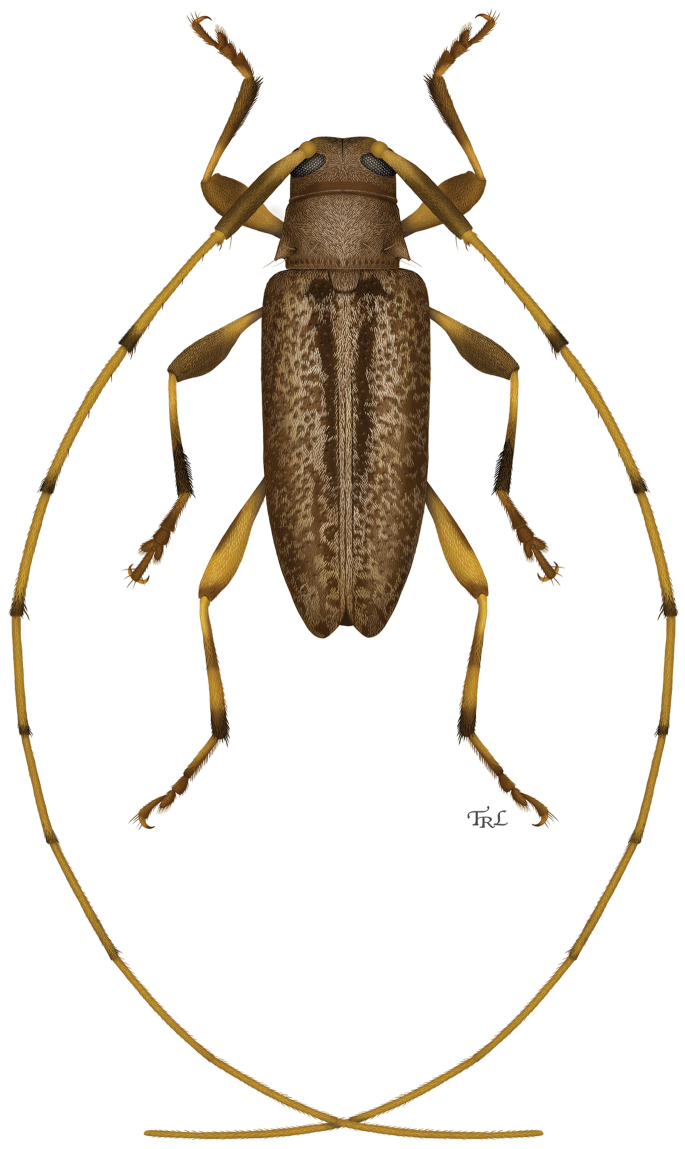
Digital painting of *Urgleptes conjunctus* sp. n. (illustrated by Taina Litwak, USDA).

Figs 4 , 8f , 11 , 18

#### Diagnosis.

The coarsely punctate elytra and narrow longitudinal darkened macula running parallel to the suture make this species readily identifiable. The mesofemora and mesotibiae of this species are distinctly darker than in other species. The antennae are most distinct in that the scape is antemedially darker than the base, and the third and fourth segments contain suberect bristle-like, apicomesal setae. Both the procoxal and mesocoxal processes are very narrow in *Urgleptes conjunctus* and barely separate the coxae.

#### Description.

*Measurements*: body length: 4.4–5.0 mm; body width: 1.5–1.8 mm; elytral length: 3.2–3.7 mm; elytral width: 0.8–0.9 mm; pronotal length: 0.8 mm; pronotal width: 1.2–1.4 mm; body length/pronotal length: 5.6–6.5; elytral length/elytral width: 4.0–4.1; pronotal length/pronotal width: 0.6–0.7.

*Head*: covered in moderately dense, appressed, pale white to golden pubescence; denser at genae, posterior margin of eye lobes and antennal tubercles. Integument mostly dark brown; slightly lighter from vertex of upper eye lobes to occiput. *Antenna*: scape basally flavous, darker submedially to apex; covered with fine, golden pubescence. Remaining antennomeres flavous; covered with moderately dense, brown setae; segments 2–7 acutely darkened at apices; segments three and four with thickened, suberect bristle-like, apicomesal setae; segments 8–11 without darkened apices. Males (most distinct in holotype) have tuft of perpendicular, thickened mesal setae at apex of fourth antennomere. *Eye*: lower eye lobe just over twice height of upper eye lobe; lobes connected by about six rows of ommatidia. *Mouthparts*: clypeus flavous; lightly pubescent basally. Labrum same color as clypeus; strongly apically rounded; with long, suberect, dark brown to gold setae; mandibles brown, darker at apical third.

*Thorax*: pronotum broadly rounded at sides to posteriorly directed, short, acute tubercles on posterior fourth; constricted along posterior fifth behind lateral tubercles; constriction demarcated with row of large, separate punctures across disc, continuing behind base of tubercles, down sides of pronotum. No distinct calli present on disc. Pronotum anteromedially covered in ashy-white pubescence extending to subapices of lateral tubercles; brown pubescence on middle of disc, extending posteriorly around posterior third of pronotum, partially obscuring row of punctures. Integument mostly brown, slightly darker at middle, extending posterolaterally and around base of lateral tubercles. Ventral surface of lateral tubercles coated in dense, appressed, golden pubescence. Prosternum smooth, impunctate, covered with uniform translucent pubescence. Prosternal process very narrow between procoxae (less than one-eighth width), barely separating them and greatly expanded posteriorly. Mesepimeron dark brown, densely coated with white pubescence, thinner at mesosternum; mesosternal intercoxal process very narrow, barely separating mesocoxae. Metepisternal integument dark brown; coated with dense, appressed, ashy-white pubescence; mostly flavous at metasternum; with moderately dense, translucent to brown pubescence. Scutellum anteriorly dark brown, slightly lighter at apex; pubescence medially expanding along posterior margin. *Elytra*: dense, irregularly spaced, distinct punctures throughout; elytral disc mottled with pallid, brown, and ferrugineous pubescence. Periscutellar region slightly swollen, darkened anteriorly flanking scutellum; posteriorly, briefly interrupted by ashy-white pubescence, resuming into single narrow longitudinal darkened macula running parallel to suture, terminating medially (in one specimen this longitudinal macula is postmedially positioned). Humeri with darkened integument and corresponding darkened pubescence extending along epipleuron. Disc mostly brown, covered in dense, appressed, ashy-white to pale pubescence. Apices subtruncate, with outer apical angle slightly more produced posteriorly than sutural angle; sutural apices dark brown, with irregular finger-like projections extending anteriorly, surrounded by mottled pale and brown pubescence. *Legs*: profemora covered in vestiture of translucent to gold setae; mostly flavous; ferruginous submedially to apices both mesally and laterally, narrowly connecting at apex; protibiae basally flavous, becoming brown submedially, covered with gold to brown setae. Mesofemora distinctly, mostly piceous, with exception of flavous base, covered in fine, translucent to gold setae; mesotibiae basally flavous, becoming dark brown apically, covered in moderately dense brown setae. Metafemora mostly flavous; with lateral dark macula submedially to apex; tibiae slightly flavous, darker at apex; both covered in vesetiture of fine gold pubescence. Tarsomeres completely dark-ferruginous with exception of slightly basally lighter first tarsomere; generally coated with short, suberect, dark setae.

*Abdomen*: ventrites covered with uniformly weakly dense, appressed, translucent to golden pubescence; integument medially light brown, becoming noticeably darker towards sides. Fifth ventrite 1.5 times length of fourth ventrite.

#### Distribution.

Endemic to Hispaniola, this rare species has been collected only in the Sierra de Baoruco mountains in August (Fig. 18).

#### Etymology.

The epithet, *conjunctus*, refers to the closely parallel longitudinal macula along the elytral suture.

#### Type material.

**Holotype** (male): **DOMINICAN REPUBLIC: Peravia Prov.**, 36 km. NW. San José de Ocoa, Aug. 9, 1979, L. B. O’Brien (USNM); **Paratypes**: same data as holotype (USNM, 2).

### Urgleptes curtipennis

Taxon classificationAnimaliaColeopteraCerambycidae

Ravin & Lingafelter sp. n.

http://zoobank.org/1FAF70B5-06B7-4874-ADBB-5ECD4D15BDC3

Figs 5 , 12 , 18

#### Diagnosis.

The proportions of this species are distinctive. The elytra are relatively much shorter compared to overall body length than all other Hispaniolan species. Further, the elytral maculations are well defined and contrast strongly from the dense, ashy, white pubescence that is otherwise present. With the exception of the pedicel and third antennomere being mostly flavous and darkened apically, all remaining antennomeres are gradually darker until the distal segments which are mostly uniform in coloration. Unlike *Urgleptes sandersoni*, *Urgleptes puertoricensis*, and *Urgleptes conjunctus*, this species has a light periscutellar region.

**Figure 12.**
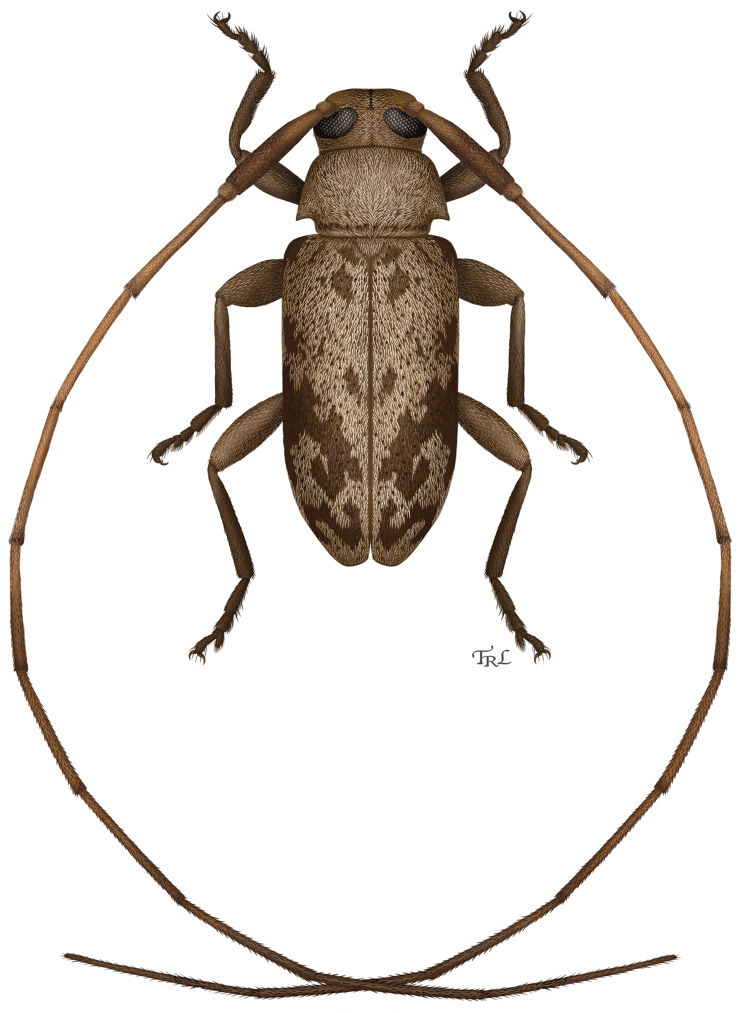
Digital painting of *Urgleptes curtipennis* sp. n. (illustrated by Taina Litwak, USDA).

#### Description.

*Measurements*: body length: 3.3 mm; body width: 1.3 mm; elytral length: 2.2 mm; elytral width: 0.6 mm; pronotal length: 0.7 mm; pronotal width: 1.1 mm; body length/pronotal length: 5.0; elytral length/elytral width: 3.5; pronotal length/pronotal width: 0.61.

*Head*: integument piceous; covered in coarse white pubescence; denser at vertex of antennal tubercles, frons, and occiput; slightly sparser on dorsal face of tubercles. *Antenna*: Scape slightly flavous at base, quickly becoming uniformly brown, covered in dense, fine gold pubescence; antennomeres three and four mostly flavous, narrowly darkened at apices; successive antennomeres mostly ferrugineous, gradually darker towards apices; with exception of slightly longer third and tenth antennomeres, segments nearly uniform in length. Antennae considerably longer than body; extending beyond elytral apices by 5–6 antennomeres; scape extending to posterior third of pronotum. *Eye*: Lower eye lobe no more than 1.5 times height of upper eye lobe; extending over half distance between antennal tubercle and frontal margin; lobes connected by 4–5 rows of ommatidia. Upper eye lobes separated by just slightly greatest width of scape. *Mouthparts*: Clypeus weakly concave, ashy-flavous, lacking distinct pubescence; labrum with a few long setae originating at base and anterior fringe of translucent setae.

*Thorax*: pronotum broadly rounded at sides to posteriorly directed, short acute tubercles on posterior fourth; constricted along posterior fifth behind lateral tubercles; constriction demarcated with row of large, separate punctures across disc, continuing behind base of tubercles, down sides. No distinct calli present on disc. Anteromedial fascia of darkened pubescence present; otherwise evenly coated in moderately dense grey-white pubescence. Integument mostly light brown, vaguely darkened at lateral tubercles to apices of spines. Prosternum smooth, impunctate, covered with sparse, gold or translucent pubescence, concentrated at posterolateral margin below lateral spines. Prosternal process narrow between procoxae (about one-eighth width) and greatly expanded behind procoxae. Mesepimeron smooth, impunctate, covered with uniform, appressed, golden or translucent pubescence. Mesosternal intercoxal process almost 2.5 times broader than prosternal process, separating mesocoxae by about one-fourth width of mesocoxa. Metasternum smooth, impunctate, covered with appressed, golden or translucent pubescence, densest at sides, thinning towards middle. Integument of ventral sclerites mostly dark brown, becoming lighter at middle. Scutellum coated in dense, appressed, ashy-white pubescence, making difficult to distinguish from elytra; broadly rounded posteriorly. *Elytra*: integument mostly dark brown; moderately punctate; almost completely obscured by uniformly dense, appressed, ashy-grey pubescence. Humeri strongly rounded with darker integument and blackened pubescence. Periscutellar region slightly swollen with two dark brown maculae; one ovate, flanking scutellum; second, larger macula set posteriorly; neither attaining suture. This region surrounded by dense, appressed, ashy-white pubescence. Elytron with sickle-shaped, post-medial, dark macula, originating at epipleuron and obliquely coiling towards middle. Posteriorly directed branches of this macula not quite attaining suture, ending at subapical, lateral margin. Darkened macula bordered by same appressed, ashy-white pubescence as anterior half. *Legs*: femora mostly ferrugineous; flavous at basal third and dorsum; covered in course white setae. Tibia flavous at base; ferruginous submedially; covered in brown, bristle-like setae. Mesofemora covered in brown to gold setae; mostly ferruginous, only flavous at base; basal third of mesotibiae flavous, dark brown submedially to apex; with postmedial concavity full of brown, bristle-like setae. Metafemora mostly flavous, slightly darker at mesal and lateral third to apices; covered in moderately dense pale gold setae. Metatibiae mostly dark brown, dark-flavous at base; covered in translucent to brown bristle-like setae. Tarsi mostly dark brown; covered in suberect, brown setae.

*Abdomen*: ventrites covered with fine, appressed, translucent pubescence, slightly thicker, gold pubescence at sides; integument light brown at middle, becoming darker toward sides. No distinct size difference in length of ventrites, with weak middle notch fringed with longer setae.

#### Distribution.

This endemic Hispaniolan species is known only from the holotype that was collected at lights on the northern face of the Sierra de Baoruco mountain range (Fig. 18).

#### Etymology.

The epithet, *curtipennis*, refers to the short elytra relative to the overall body length.

#### Type material.

**Holotype** (male): **DOMINICAN REPUBLIC: Independencia Prov.**, Sierra de Baoruco, Rabo de Gato, 18°17.962'N, 71°35.811'W, 490 m, 14–15 Dec 2014, S. W. Lingafelter, UV/MV lights (USNM).

### Urgleptes marionae

Taxon classificationAnimaliaColeopteraCerambycidae

Ravin & Lingafelter sp. n.

http://zoobank.org/72996267-F241-41D2-94FD-10C65EA0351C

**Figure 13.**
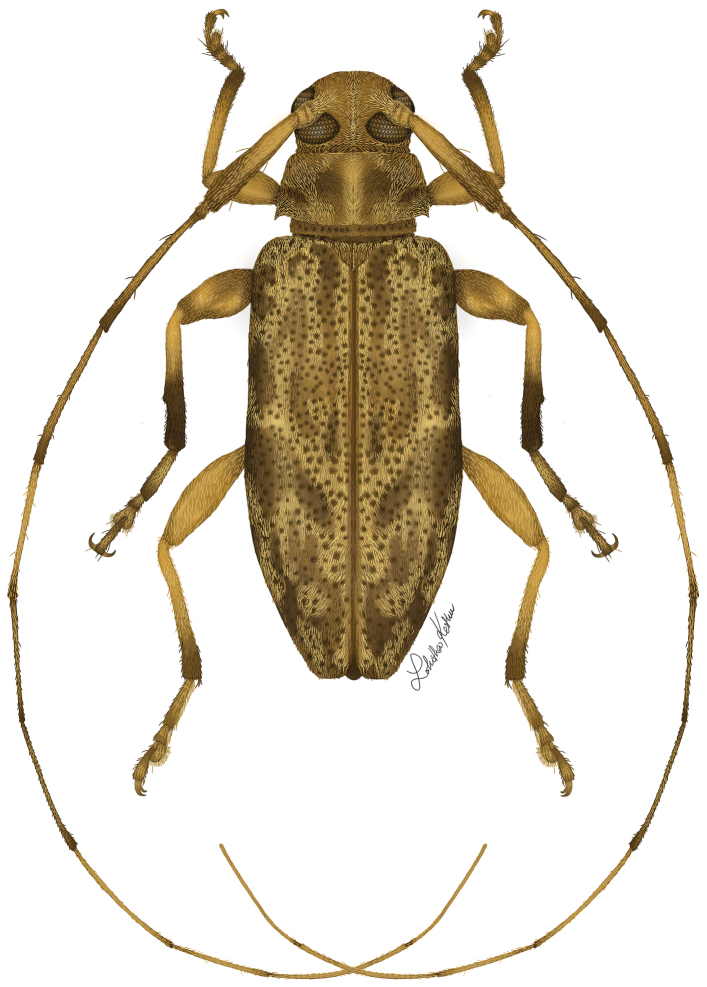
Digital painting of *Urgleptes marionae* sp. n. (illustrated by Lohitha Kethu, Virgina Commonwealth University).

Figs 6 , 8g , 13 , 19

#### Diagnosis.

The elytra of this species each have an oblique postmedial macula extending anterolaterally from near the suture to middle lateral margin, somewhat similar to *Urgleptes sandersoni*, *Urgleptes puertoricensis*, and *Urgleptes curtipennis*. However *Urgleptes marionae* is unique in that faint, longitudinal, darker maculae are present on the elytra, extending posteriorly from the base, much less defined than in *Urgleptes conjunctus*, and there are three irregular, longitudinal fasciae of pale pubescence. The femora are flavous and densely covered in mostly golden pubescence, with the pro- and mesofemora each containing a medially darkened ring along the dorsal and mesal margins. The mesocoxal process in *Urgleptes marionae* is much broader than in *Urgleptes conjunctus*.

#### Description.

*Measurements*: body length: 4.8–6.3 mm; body width: 1.7–2.2 mm; elytral length: 3.5–4.9 mm; elytral width: 0.8–1.1 mm; pronotal length: 0.8–0.9 mm; pronotal width: 1.4–1.7 mm; body length/pronotal length: 6.4–6.7; elytral length/elytral width: 4.2–4.4; pronotal length/pronotal width: 0.5–0.6.

*Head*: integument dark brown, slightly lighter at antennal tubercles; with exception of mostly glabrous genae, covered in dense, appressed, ashy-white pubescence, denser at posterior eye margin. *Antenna*: scape basally flavous, dark brown submedially to apex; covered with fine, translucent pubescence. Antennomeres 3–6 flavous, darkened apically, with fine gold to brown pubescence; apical segments without dark apices, uniformly light brown; basal segments with thickened, suberect bristle-like, apicomesal setae. *Eye*: lower eye lobe just over twice height of upper eye lobe; lobes connected by about seven rows of ommatidia. *Mouthparts*: clypeus light-flavous; lacking pubescence. Labrum flavous; apically fringed with translucent setae, and posteriorly with long, suberect, dark brown setae; mandibles light-ferruginous, darker at apices.

*Thorax*: pronotum broadly rounded at sides to posteriorly directed, short, narrow, acute tubercles on posterior fourth; constricted along posterior fifth behind lateral tubercles; constriction demarcated with row of large, separate punctures across disc, continuing behind base of tubercles, down sides of pronotum. No distinct calli present on disc. Pronotum dark brown; anteromedially covered in appressed, ashy-white pubescence extending posteriorly; flanked by dark, irregular fascia, surrounded by ashy-white pubescence. Prosternum smooth, impunctate, covered with uniform ashy to golden pubescence. Prosternal process narrow between procoxae (less than one-sixth width) and greatly expanded posteriorly. Mesepimeron dark brown, densely coated with ashy to golden pubescence, thinner at mesosternum; mesosternal intercoxal process moderately broad between mesocoxae, about twice width of prosternal process between procoxae. Metepisternal integument dark brown; coated with dense, appressed, ashy to golden pubescence; light brown at metasternum; with fine translucent pubescence. Scutellum uniformly dark brown; covered in ashy-white pubescence. *Elytra*: light brown; covered with moderately dense punctures, partially obscured by dense, mottled ashy-white to brown pubescence. Periscutellar region slightly swollen, with dark, longitudinal, posteriorly directed, incomplete maculae terminating basally and medially. Base of elytron with dark, lateral, posteriorly directed macula interrupted into thirds; terminal macula posteriorly slanted to, but not attaining sutural margin. Sutural apices dark, with irregular finger-like maculae extending anteriorly, connecting to obliquely transverse macula; surrounded by dense, uniform, ashy pubescence. Apices subtruncate, with outer apical angle slightly more produced posteriorly than sutural angle. *Legs*: pro- and mesofemora covered in translucent to gold setae; integument mostly flavous; light brown submedially to apices both mesally and laterally, narrowly connecting at apex. Protibiae basally flavous, becoming brown submedially, covered with gold to light brown setae; mesotibiae basally flavous, dark brown submedially, covered with dark brown, dense, bristle-like setae. Metafemora covered with fine translucent to gold pubescence; mostly flavous, with vague, lateral, postmedial, light brown spot; apex slightly darker; tibiae basally flavous, dark apically, with brown pubescence. Tarsomeres basally flavous, dark apically; generally coated with short, suberect, dark setae.

*Abdomen*: ventrites covered with fine vestiture of appressed, translucent to golden pubescence; integument dark flavous; fifth ventrite brown, one and one-half the length of the fourth ventrite. Last ventrite weakly concave and fringed with long golden pubescence.

#### Distribution.

Endemic to Hispaniola, this species has been collected just east of the Haitian border in the Sierra de Neiba mountains and in the northeast Cordillera Central (Fig. 19).

#### Etymology.

We are pleased to name this species after Grace Natalie Marion (1925–2014), grandmother of the first author. The epithet is a noun in apposition.

#### Type material.

**Holotype** (male): **DOMINICAN REPUBLIC: Monseñor Nouel Prov.**, Cabo Vito 19°01.165'N, 70°31.197'W, 4 July 2004, beating, C. J. Micheli (USNM); **Paratype: Elías Piña Prov.**, Sierra de Neiba, 9.3 km SW Hondo Valley, 1901 m, 18°41'31"N, 71°47'03"W, montane forest, *Podocarpus* sp., UV light, 30 April 2006, J. Rawlins, J. Hyland, R. Davidson, C. Young, D. Koenig, J. Fetzner (CMNH).

### Urgleptes obliteratus

Taxon classificationAnimaliaColeopteraCerambycidae

Ravin & Lingafelter sp. n.

http://zoobank.org/2310C234-C85F-4F47-9C5D-3A707F18FC79

**Figure 14.**
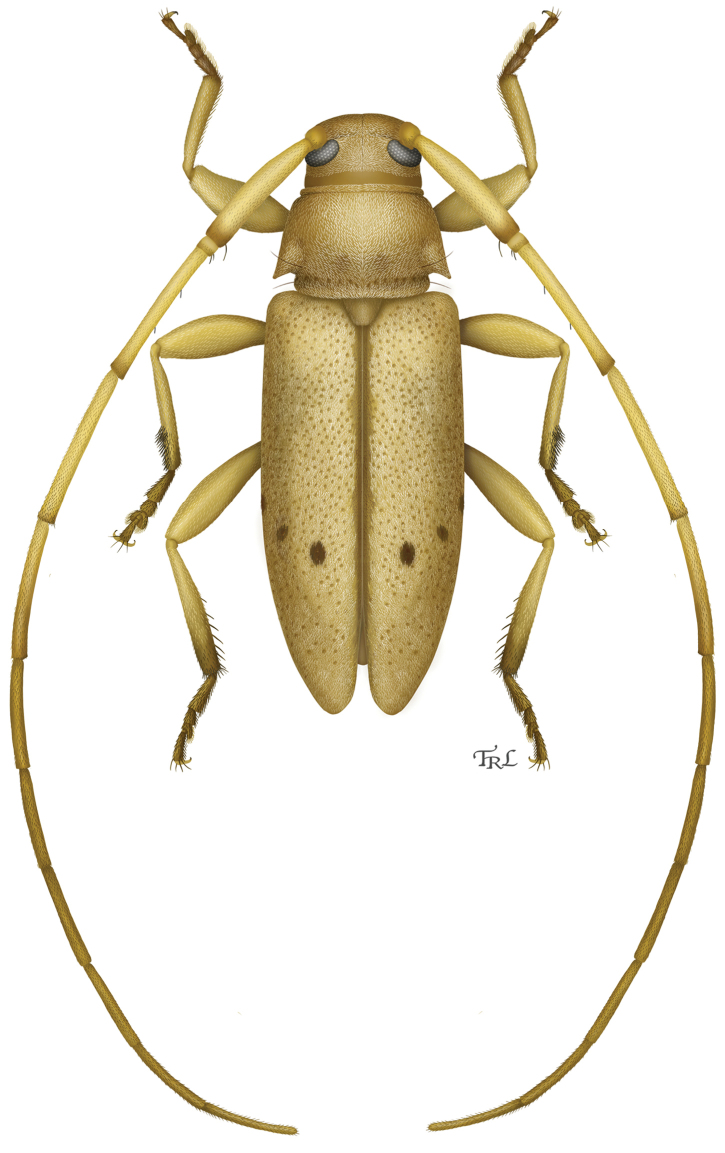
Digital painting of *Urgleptes obliteratus* sp. n. (illustrated by Taina Litwak, USDA).

Figs 7 , 8d , 14 , 19

#### Diagnosis.

This species is easily identified by the three dark, circular elytral maculae, originating mediolaterally and extending toward the suture. This dark circular pattern is repeated on the pronotum which usually contains two posteromedial spots, in some specimens, an additional two are located at the base of the lateral tubercles. The elytral suture is narrowly dark brown. Otherwise, the integument is mostly uniform in color, although in *Urgleptes obliteratus* it is much paler yellow or flavous than in *Urgleptes charynae*. Unlike other species, the scape is uniformly pale, with antennomeres light, gradually becoming darker at apices. The femora are also mostly pale, with the tibiae usually darker postmedially.

#### Description.

*Measurements*: body length: 3.6–4.8 mm; body width: 1.3–1.7 mm; elytral length: 2.5–3.3 mm; elytral width: 0.6–0.8 mm; pronotal length: 0.7–0.9 mm; pronotal width: 1.0–1.6 mm; body length/pronotal length: 5.2–5.4; elytral length/elytral width: 4.0; pronotal length/pronotal width: 0.5–0.6.

*Head*: covered in uniform pale to golden pubescence, denser at posterior margin of eyes and inner eye notch margin. *Antenna*: scape extending to posterior fourth of pronotum; pale-flavous, weakly darker apically; covered in fine, pale to golden pubescence. Remaining antennomeres pallid, darkened apically; covered in brown pubescence, with thickened bristle-like setae at apices; mesal surface of third and fourth segments of most specimens usually with thickened setae. Antennae extending beyond elytral apices by approximately five segments. *Eye*: lower eye lobe about 2.5 times height of upper eye lobe; lobes narrowly connected by about 5 rows of ommatidia in most specimens. Upper eye lobes separated by twice width of upper eye lobe. *Mouthparts*: clypeus pallid; lacking distinct pubescence. Labrum pallid; anteriorly fringed with long, golden setae; and basal, longer, suberect, dark brown setae. Mandibles light brown, distinctly darkened on apical halves.

*Thorax*: pronotum broadly rounded at sides to posteriorly directed, short, acute tubercles on posterior fourth; constricted along posterior fifth behind lateral tubercles; constriction demarcated with row of large, separate punctures across disc, continuing behind base of tubercles and down sides; no other distinct punctures visible. Integument mostly light brown; coated in moderately dense vestiture of white pubescence. Pronotal disc with dark anterolateral and posteromedial fasciae. No distinct calli present on pronotal disc. Prosternum impunctate; covered in moderately dense white pubescence. Prosternal process extremely narrow and greatly expanded posteriorly; procoxal cavities nearly touching. Mesepimeron toward metepisternal margin coated with dense, appressed, white pubescence becoming thinner ventrally. Mesosternal intercoxal process about 3–4 times broader than prosternal process. Integument of ventral sclerites mostly light brown, with margins of coxal cavities darkened. Scutellum dark brown; with white, longitudinal fascia. *Elytra*: moderately, densely punctate; elytral disc mostly pale-orange with suture dark brown from scutellum to apices. Periscutellar region slightly swollen; coated in white to translucent pubescence extending throughout disc; epipleuron slightly darker than surrounding region. Elytron with three, dark, distinct, circular maculae, originating mediolaterally, obliquely extending toward, but not reaching suture. Some specimens with faint, irregular macula extending anteriorly from apex but not reaching circular maculae. Elytral apex subtruncate, with outer apical angle slightly more produced posteriorly than sutural angle. *Legs*: femora mostly pallid; uniformly covered with golden to translucent pubescence. Protibiae uniformly pallid; meso- and metatibiae darker apically with thickened, bristle-like setae. Mesotibiae with dorsal concavity apically that is lined with dark, bristle-like setae. Tarsomeres dark brown; coated with long, suberect, dark setae.

*Abdomen*: ventrites covered with fine, appressed, white pubescence; integument mottled brown, posteriorly lighter, apical margin of all ventrites slightly lighter. Fifth ventrite one and a half times longer than fourth. Last tergite strongly narrowed and projecting beyond last ventrite.

#### Distribution.

Endemic to Hispaniola, this species has been collected only in low elevation (less than 700 meters) coastal areas of the country (Fig. 19) from May through December.

#### Etymology.

The epithet, *obliteratus*, refers to the nearly absent maculae on the elytra, unlike most other species of the genus.

#### Type material.

**Holotype** (female): **DOMINICAN REPUBLIC: Pedernales Prov.**, 25 N of Cabo Rojo, 750 meters 18°06.769'N, 71°37.245'W, beating, 11 December 2014, S. W. Lingafelter (USNM); **Paratypes: DOMINICAN REPUBLIC: María Trinidad Sánchez Prov.**, Río San Juan, 19°37'17"N, 70°7'45"W, 20 July 2008, Julien Touroult (JTPC, 2); **Puerto Plata Prov.**, South of Pico Isabel de Torres, El Cupey Rd., 258 m, 19°45.214'N, 70°43.6464'W, 30 July 1999, Ivie & Guerrero (WIBF, 2); **San Pedro de Macorís Prov.**, 12 km W of San Pedro de Macorís, 5–19 May 1985, E. Giesbert (FSCA).

### Key to the species of *Urgleptes* of Hispaniola

**Table:** 

1	Scape with flavous subapical spot between narrow, darkened apex and broadly darkened postmedial region (Fig. 8a); elytral periscutellar area darkened and contrasting with surrounding region; elytra with obliquely transverse, postmedial dark macula; femora mostly flavous with dark macula both mesally and laterally, most prominent on mesofemora	***Urgleptes sandersoni***
–	Subapical spot absent from scape (Fig. 8b-d); other characters variable	**2**
2(1)	Scape mostly uniformly colored (Fig. 8c-d); elytral integument mostly uniformly colored (Fig. 10, 14)	**3**
–	Scape basally flavous (Fig. 8b), darkened apically; elytral integument mottled light and dark (Figs 1, 11, 12, 13)	**4**
3(2)	Integument tawny brown; pronotum with dense, white pubescence concentrated on posterior constriction behind lateral tubercles; elytral suture of same color as disc; elytra with postmedial patches of dense white pubescence extending across disc; slightly darkened integument underneath patches (Fig. 10)	***Urgleptes charynae* sp. n.**
–	Integument pale; pronotal disc with dark anterolateral and posteromedial fasciae; elytra with darkened suture from scutellum to apices; elytra with three, dark, mediolateral, circular maculae, obliquely extended toward suture (Fig. 14)	***Urgleptes obliteratus* sp. n.**
4(2)	Elytra with longitudinal, dark maculae, extending posteriorly from base (Figs 11, 13)	**5**
–	Elytra without longitudinal macula; with dark, transverse, postmedial macula extending obliquely from lateral margin to suture (Figs 1, 12)	**6**
5(4)	Elytra with faint, posteriorly directed, incomplete maculae at base. Apices of elytra with dark, finger-like maculae extending anteriorly, connecting to obliquely transverse macula. Punctures on elytral disc mostly obscured beneath appressed pubescence. Pro- and mesofemora with dark, lateral and mesal maculae (Fig. 13)	***Urgleptes marionae* sp. n.**
–	Each elytron with single narrow longitudinal darkened macula running parallel to suture, terminating medially; elytra with moderately dense, distinct punctures, clearly visible beneath pubescence; oblique, transverse macula absent; mesofemora distinctly darker than pro- and metafemora (Fig. 11)	***Urgleptes conjunctus* sp. n.**
6(4)	Periscutellar region light; distinctly covered in dense, appressed, ashy pubescence with well-defined, dark, postmedial macula obliquely coiled from lateral margin to middle. Most antennomeres (with exception of third antennomere that is mostly flavous and apically darkened) gradually darker; distal segments mostly uniform in coloration (Fig. 12)	***Urgleptes curtipennis* sp. n.**
–	Periscutellar region dark with postmedial zigzag fascia obliquely extending towards suture; antennomeres 3–7 flavous, dark annulate at apices (Fig. 1)	***Urgleptes puertoricensis***

**Figure 15.**
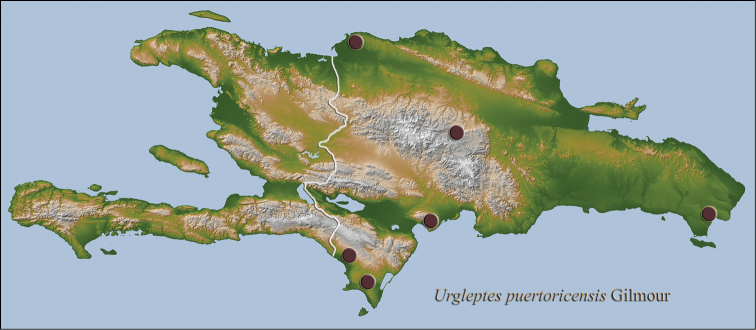
Hispaniolan distribution map for *Urgleptes puertoricensis* Gilmour.

**Figure 16.**
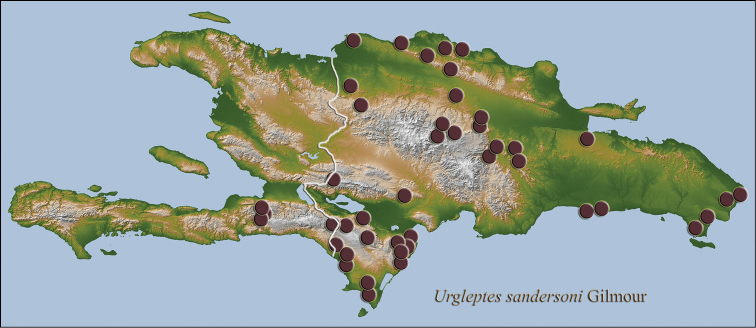
Hispaniolan distribution map for *Urgleptes sandersoni* Gilmour.

**Figure 17.**
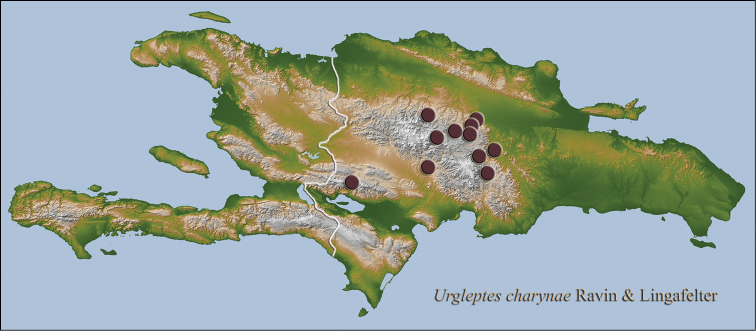
Hispaniolan distribution map for *Urgleptes charynae* sp. n.

**Figure 18.**
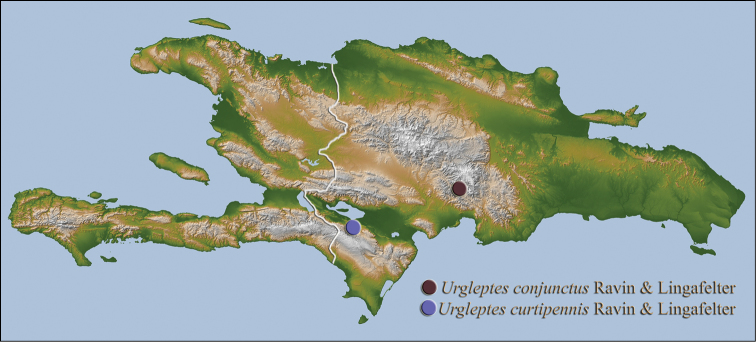
Hispaniolan distribution map for *Urgleptes conjunctus* sp. n. and *Urgleptes curtipennis* sp. n.

**Figure 19.**
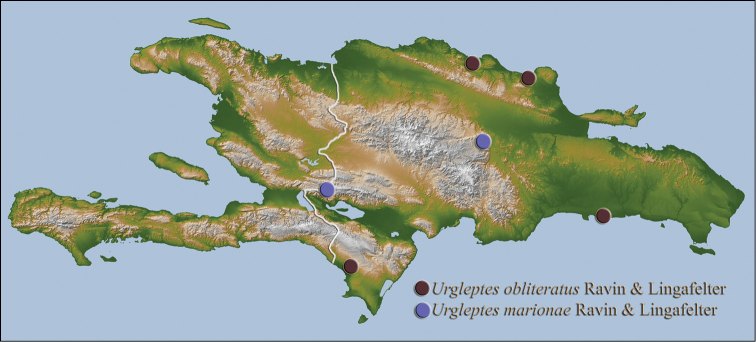
Hispaniolan distribution map for *Urgleptes marionae* sp. n. and *Urgleptes obliteratus* sp. n.

## Supplementary Material

XML Treatment for Urgleptes puertoricensis

XML Treatment for Urgleptes sandersoni

XML Treatment for Urgleptes charynae

XML Treatment for Urgleptes conjunctus

XML Treatment for Urgleptes curtipennis

XML Treatment for Urgleptes marionae

XML Treatment for Urgleptes obliteratus
